# From food to cash assistance: rethinking humanitarian aid in Yemen

**DOI:** 10.1186/s41018-022-00119-w

**Published:** 2022-04-23

**Authors:** Moosa Elayah, Qais Gaber, Matilda Fenttiman

**Affiliations:** 1 Doha Institute for Graduates Studies, Doha, Qatar; 2 Centre for Governance and Peacebuilding Nijmegen, Nijmegen, The Netherlands

**Keywords:** Humanitarian aid, War, Food Assistance, Cash assistance, Civil protection, Yemen

## Abstract

Humanitarian aid (HA) is needed in Yemen to cope with the worst humanitarian crisis in the world. However, current practices of distributing aid in the form of food have not had the desired effect; conflict has continued, and war economies are thriving as a result. Thus, this paper proposes the idea of cash assistance as an alternative form of HA. Our empirical analysis of HA in Yemen shows that cash assistance is a more effective and efficient way of distributing aid in comparison to food aid. This is due to food aid being vulnerable to looting by the conflicting factions, enabling funds to be inadvertently captured into the highly problematic war economy dynamic. In comparison, cash assistance targets a wider scope of peoples’ necessities, in a more efficient and effective delivery approach that can be easily tracked by the donors. Cash aid can either be unrestricted, restricted, or conditional. The former allows cash transfer, the second enables vulnerable people to purchase items depending on their needs with vouchers, and the latter links the money distributed with performing a certain task. This allows communities to improve and develop, and it enables individuals to build up their skill sets and have a source of income. This is particularly important for NGOs who are unable to provide the conditions needed to bear their success. The Yemeni people have lost trust in the warring factions, as well as local and international NGOs, due to the lack of effectiveness of current methods of HA distributions due to looting and the risks associated with reaching vulnerable people. Therefore, it has become imperative to restore donor direct HA delivery by providing cash aid as a superior means of food aid in Yemen, to ensure the effectiveness of HA and to improve the lives of those who are suffering, in the long term (This article builds on previous scholarship; see Elayah, M., & Fenttiman, M. (2021). Humanitarian Aid and War Economies: The Case of Yemen. *The Economics of Peace and Security Journal*, *16*(1). This article provides a nuanced and high-quality examination of the conduct of armed groups in Yemen. The analysis of HA in this article “supports the view that it is a significant source of funding for armed groups and consequently that it plays a huge part in allowing the war economy in Yemen to thrive”. Warring groups are often looting HA to distribute it based on partisanship and to sell it on the black market to finance the war effort. They have also attempted to block HA to try and gain control over the humanitarian campaign and receive a cut of the billions of dollars given in foreign assistance. In many cases, HA is distributed through local NGOs that were established by the groups to attract international funds. Others were pre-politicized NGOs that channeled funds to specific regions or particular groups for political and military advantage. It is clear from our analysis that the ability of NGOs to use HA effectively and deliver it to those who deserve it is very limited. NGO’s actions can end up expanding the war economy rather than reducing the effects of war on the poor. Distrust in international bodies and in local and international NGOs has become extremely high among those affected by the war” (Elayah, M., & Fenttiman, M. (2021: p 59)).

## Introduction

Since the start of the Yemeni conflict in 2015, the situation has been deteriorating. Yemen is the biggest humanitarian crisis in the world since World War II. Around three million people have fled their homes, while two million are still internally displaced, and one million houses have been destroyed. More than twenty-two million people, ten million of which are children, are in dire need for humanitarian assistance. Furthermore, about eight million Yemenis have lost their livelihoods. A huge percentage of Yemeni people used to work for the public sector, but due to economic deterioration, more than one million government employees have stopped receiving wages (UN 2019; Bandsom 2018). The economy has been continually declining since the start of the war, Gross Domestic Product (GDP) has decreased by 40%, and the Yemeni Riyal has lost more than 75 %of its value, consequentially increasing the prices of imported goods exponentially (UN 2019; World Bank 2018). According to a World Bank report, the private sector in Yemen has been successful in maintaining the supply of goods, but the small demand and purchasing power incapacity have been major obstacles and these factors are driving Yemen towards a famine crisis (World Bank 2018).

Non-governmental Organizations (NGOs) play a major role in the development of societies as they are the actors entrusted by donors to deliver aid programs to the beneficiaries. NGOs are tasked to design and implement the most efficient and effective projects, aligned with the respected communities’ needs and growth objectives, to further social and political goals. In conflict countries such as Yemen, aid programs have shifted towards relief work, offering emergency humanitarian assistance by distributing everyday commodities, mainly food. Yet offering food aid has proved inadequate in similar situations (Clay 2005; Barrett and Maxwell 2005). In recent years, delivering those supplies in Yemen has become less effective due to corruption, ununified aid policies, de facto-state influence (Governing bodies not officially recognized), and the fragility of public institutions (Elayah 2016). Those criticisms came with an increased number of financial and non-financial public corruption scandals in the foreign aid industry, including the global NGO sector (Kiley et al. 2019; Hoelman 2009; Gibelman and Gelman 2001). Food aid may not be suitable all the time, and it has proven to be less efficient and effective than cash-based assistance (Castillo 2021; Vogel et al. 2021)^1^. Consequentially, International Non-governmental Organizations (INGOs) recognized that they had to innovate and diversify the delivery mechanisms of aid in conflict areas. INGOs started to provide aid to people in emergencies in the form of cash; either conditional (cash for work), unrestricted (direct cash transfer), or restricted (through vouchers). Moreover, the cash transfer is aimed to empower people to decide on their basic needs, as well as making it easier for them to access. Existing literature on cash-based assistance presents positive results, but the impact is still unrecognized in countries of conflict because organizations have been hesitant to implement this system (Harvey 2005).

This paper aims to compare the impact of cash transfer and food humanitarian assistance during emergency and conflict situations in Yemen. This paper examines cash distribution projects implemented in Yemen, a country with a quasi-total state failure facing multiple humanitarian crises. The study discusses cash intervention utilization, impact, and limitations, in contrast to the food aid. This paper aims to contribute to the existing literature by investigating and describing the experience of the Yemen Cash Project, hoping to offer donors and NGOs insight on whether cash transfer is suitable in conflict areas and in Yemen specifically.

In order to understand the challenges and inadequacies fronting the aid distribution process in Yemen, this study explores primary research, using a purposive sample to enrich this study with empirical data. Discussing the challenges facing NGO’s aid distributions in Yemen and various reasons behind food-aid diversion, the findings attempt to explain how cash-based interventions can overcome the limitations of the currently used aid mechanism, and the way in which this solution should be implemented.

### Cash transfer as an aid alternative: theoretical elements

In 1864, the International Committee of the Red Cross (ICRC) became the world’s first official international humanitarian organization aimed at providing HA to civilian and military victims of conflict. HA today provides relief across an array of contexts, but originally, it was almost exclusively supplied to conflict areas (Paulmann 2013). To analyze the history of HA, Barnett (2011) suggests that there are three distinct ages of humanitarianism, “an imperial humanitarianism, from the early nineteenth century through World War II; a neo-humanitarianism from World War II through the end of the Cold War; and a liberal humanitarianism, from the end of the Cold War to the present”.

As technologies developed during the “imperial humanitarianism” period so did the human costs of conflict; “armed conflict was becoming less and less a chivalrous jousting contest for the few, and more and more a mass slaughter”^2^. Individuals began imagining new ways to relieve human suffering, through a Eurocentric idea of the international community. Before World War I, the work of the ICRC was largely a small affair but when the Treaty of Versailles led to the formation of the League of Nations (later renamed the UN) to protect vulnerable populations and encourage peace, the states became increasingly involved in humanitarian action (Barnett 2011, 29).

In the “neo-humanitarianism” period following World War II, the number of humanitarian and intergovernmental agencies grew rapidly. In the 4 years succeeding the war, over 200 new NGOs were formed globally and the UN grew in both size and significance (Rysaback-Smith 2016). The UN’s progress led to the conclusion of the Universal Declaration of Human Rights in 1948, and the formation of many organizations under the auspices of the UN such as the WHO (1948), UNICEF (1946), and UNHCR (1950) (Paulmann 2013). Kent (1987, 36) argued that this was a turning point for HA as “it was only in the midst of World War II that governments began to fully appreciate the need for greater international intervention in the plight of disaster-stricken people”. The relief effort became increasingly international due to advancements in technology, transport, and communication, and this resulted in a shift of focus from Europe to less developed countries, thus laying the foundation for the international HA network we have today.

Barnett (2011) coined the third age “liberal humanitarianism” due to the global efforts to create a liberal peace. The proliferation of NGOs in the 1980s resulted in an increase in public awareness through increased media coverage and advertising campaigns. The 1984 televised video footage of starving children in Korem, Ethiopia, and the 1985 Live Aid concert, the first big, televised concert to raise money for HA, helped to highlight problems of poverty globally (Westley 1991). However, the modern concept and system of HA—characterized by humanity, neutrality, impartiality, and independence—has only become a commonplace since the 1990s when these basic principles were established by the UN General Assembly in 1991 (Elayah 2021; Rysaback-Smith 2016). Over the past three decades, there have been radical advancements in medicine, delivery systems, and logistical capacities enabling humanitarian action to become more efficient. However, the new global environment has also created many dangers and challenges. Following the 9/11 attacks, there was a major concern with the dangers that failed states posed to themselves and others and saving them became a matter of international human security.

The main challenge conflict zones they are facing is how to accommodate universalism in a world of diversity, since globalization is clashing with traditional values around the world leading to the development of war economies (Barnett 2011). In many cases, these war economies have caused governance systems to become weak and have resulted in their collapse, thus leading to long-term issues that result in the recurrence of conflict (Pugh et al. 2005). In Cambodia, natural resources such as timber and rubber have been a large source of funding for armed groups. Afghanistan is also the world’s largest opium producer and center for arms dealing, which has a million-dollar trade revenue per year from smuggling these commodities in Pakistan from Dubai (DeLozier 2019). Furthermore, while HA is desperately needed, international and humanitarian organizations are in danger of being perceived as siding with the West. This was the case in the August 2003 Baghdad bombing, now known as the UN’s 9/11, which killed the Undersecretary-General Sergio Viera de Mello and 21 others (Benthall 2003; Donini 2004; Hyder 2007).

The number of people suffering in conflict environments is increasing despite the huge developments and progress within the HA field since the 1990s. The United Nations Office for the Coordination of Humanitarian Affairs (OCHA) estimated that in 2020, 167.6 million people around the world would require HA (~ 1 in 45 people globally) (United Nations Office for the Coordination of Humanitarian Affairs (OCHA) 2020), and Kim (2017) estimates that by 2030, 50% of the global poor will live in areas where conflict and fragility are rife. In Nigeria, South Sudan, Somalia, and Yemen, alone, the number of people suffering from famine reached twenty million in 2018. This figure has increased at such a high rate that the funding gap has risen from around US $2.5 billion in 2009 to around US $13 billion in 2019 (United Nations Office for the Coordination of Humanitarian Affairs (OCHA) 2020). Thus, the current method of HA application to improve crisis situations has produced much skepticism and cynicism internationally, since humanitarian organizations have often failed to achieve goals and help endangered civilians and aid workers alike (Terry 2011; Findley 2018).

Furthermore, the warring parties are impeding the delivery of HA in Yemen as part of a larger plan to gain power and control in the conflict, while making money from looting and selling HA on the black market and providing preferential treatment to the communities supporting them (Elayah and Fenttiman 2021). The war economy began from the loyalist militias for all fighting parties dealt with the country’s capabilities and stockpiled the HA, which it harnessed to finance its wars and enrich its loyalists. For example, the Houthi group used oil revenues, extortion, and levies imposed on citizens and merchants, even at the level of kidnappers and their families. Their control begins at the ports, which facilitate their imposition of levies and taxes on shipments, as well as enabling them to seize HA coming into the country (Elayah and Fenttiman 2021). This has resulted in Yemen turning into a black hole for HA, where supplies do not go to those who need it, but on the contrary, it reaches through the lobbies of warlords who play the most important role in prolonging and sustaining a war, by financing it (Elayah and Verkoren 2020).

In the current climate, it seems that no amount of HA will offset the societal collapse, high food prices, and consequently the amount of suffering that the war has produced. The UN claims that the weaponization of HA highlights a “collective failure and collective responsibility” of all actors involved, since the political unrest has been driven by the efforts of various entities, both governmental and non-governmental, who enable the war economies to thrive (UNHRC, 2019). Thus, it has become vital to seek out new approaches to prevent the situation from deteriorating even more, and the section below will explain the theoretical elements of our proposed solution, cash aid.

Implementing cash transfer projects is under-utilized in the NGO sector, as it amounts to only 17.9% of all forms of HA (CaLP 2020b). Nonetheless, it is becoming a more effective alternative in conflict areas and emergency settings, and by 2018, the total aid in form of cash reached US $4.7 billion^3^, a rise from US $1.2 billion in 2014 (Overseas Development Institute (ODI) and the Center for Global Development (CGD) 2015). The cash-based interventions are being adopted by various organizations in Yemen including the World Food Program (WFP) whose previous explicit food-aid mission was altered to include offering “food assistance”. Food assistance programs offer cash-based aid in different forms including vouchers that can be replaced with what the beneficiaries need at selected stores (Pongracz 2015). Vouchers are known to be restricted because there is a limitation on how and where they can be used. In comparison, cash transfers are unrestricted because they can be spent how the recipient chooses (The Cash Learning Partnership 2020a).

The first recorded use of HA cash transfers was during the Franco-Prussian War of 1870–1871. The American Red Cross used this method to set up micro-enterprise projects for wounded soldiers recovering from the war. There has been a lot of success since. For example, during the tsunami crisis in 2004, governments decided to use cash-based aid as a replacement for food aid, enabling people to rebuild, rent, and regenerate their livelihoods (Adams and Winahyu 2006; Harvey 2007). In 2010, a flood hit Pakistan and the government distributed US $1.7 million in debit cards as an aid to help people suffering from the disaster (Overseas Development Institute (ODI) and the Center for Global Development (CGD) 2015). The same approach is used among some INGOs operating in conflict areas, to permit the vulnerable to decide what to buy based on their individual needs (Mattinen and Ogden 2006). This form of assistance prevents people from reselling the food-aid supplies to generate an income and buy other necessities “as, for example, 70% of Syrian refugees in Iraq have reportedly done” (Mattinen and Ogden 2006).

Increasing an individual’s spending capacity enables access to a wider option of life priorities, in non-degrading ways, including treatment, clothes, housing, and educational institutes (Mattinen and Ogden 2006; UN 2019). Reducing negative conditions on a national level, such as food limitations, poverty rates, child labor, and armed group expansion (Lehmann and Masterson 2014), revives the economy and encourages small businesses. This is an integral step towards a sustainable future (Mattinen and Ogden 2006) as it aligns the agendas of international humanitarian assistance with the needs of the local people.

Another form of cash-based relief is vouchers which the World Food Program employed on the one million Syrian refugees they help in Lebanon (Pongracz 2015). In essence, the vouchers enable people to purchase items that they need from stores, but they have created another inflation dilemma. For instance, in Lebanon where the World Food Program offers cash help to one million Syrian refugees in vouchers, due to the vouchers being valid at only certain stores, those vendors turned into monopolies. This led to increasing commodity prices every month, resulting in vouchers losing around a million each time the prices increased (Pongracz 2015).

For the NGO sector, cash-based interventions are more efficient and cost-effective compared to distributing food. Studies in Yemen and three other countries concluded that offering cash instead of food will help NGOs give assistance to 18% more beneficiaries at the same operational cost. Another report from Zimbabwe showed the superiority of cash interventions on the household income, as it stated that every dollar given directly as aid increased the income by $2.59, while food-aid intervention only increased the income by US $1.67 (Concern Worldwide 2011). Operational costs are lower for cash-aid programs than for delivering food aid because this method requires less importation, local transport, storage, human resources, and logistics (UN 2019). On the other hand, INGOs can deliver cash programs without the need for multiple local Ngo’s involvement, which consequently will decrease the influence of “twilight groups” working with hidden agendas. It lowers the risk of commodity distribution being diverted or resold by these groups on secondary markets to keep funding their own agendas. Furthermore, cash-based interventions offer digital transactions, a less risky method of distribution compared to food aid and allows equitable access and distribution of HA supplies. According to the UK National Audit Office 2011, transferring cash and assets to the poor report, cash transfers using electronic methods will increase the safety, cost-effectiveness, and reduce fraud risks compared to food distributions (UK National Audit Office 2011). According to the Overseas Development Institute (ODI) and the Center for Global Development (CGD) (Overseas Development Institute (ODI) and the Center for Global Development (CGD) 2015), this was successfully tested by the World Food Program in Ethiopia, where the cash-aid project was between 25 and 30% more efficient than the existing food-aid projects. Cash aid has also proven to be effective within unstable countries such as in Somalia, where cash aid was delivered to one and a half million vulnerable civilians in 2011. The cashless transaction also offers the donors a transparent means to trail the funds until they reach their recipients, to prevent fraud and exploitation and to reduce the amount of HA being lost to the belligerent actors.

Nonetheless, if cash aid is not accompanied by accurate monitoring measurements, then it can fall into the corruption trap, and it is likely to be a more attractive commodity than food aid. Moreover, if the proper research was not done prior to the implementation process the new cash supply injected into the market can negatively affect the economy.

Reprinted Table 1 from (Allahi et al. (2019) “Cash-based interventions to enhance dignity in persistent humanitarian refugee crises: a system dynamics approach & (CaLP 2020b)—The Cash Learning Partnership.” explains the advantages and disadvantages of implementing the cash-aid projects.

**Table 1:** Cash as AID advantages and disadvantages

Cash as aid	
Advantages	Clarification
Maintain people’s dignity and increase their basic needs access	Empowering people, to decide on their different basic needs and buy what matters to them based on their situations
Enhance housing conditions	Giving people the chance to rebuild their houses, rent temporary places
Financial security	People can save up some money, pay back debts, and start mini projects
Better food diet	People can use the money to buy different forms of food
Better living conditions could decrease poverty rates	People who cannot work will have an income, such as elderly and disabled people. On the other hand, people with low skills can make an income working for cash projects. This increases their skills and enables them to find other job opportunities
Increases health and education rates	People now will have extra money to spend on health and education. This will give more children the opportunity to go to school.
Engages the private sector	High demand together with individuals having cash, provides an opportunity for the private sector to supply missing commodities
Decreases the INGO’s operational costs	Cash transition programs need less logistics cost on transportation, storage etc.
Better conditions for children	Family income will decrease the need for children to work. They will have time to study, play, and live a normal life
Digital cash is easier to track and distribute	Decreasing the risk of corruption and supplies being controlled by “twilight groups” and resold into secondary markets, to fund their own agendas
Disadvantages	Clarification
Economy stagnation and dependency	People may get used to receive aid money and supplies and stop looking for different income sources (mainly with direct cash aid)
Women, children, and elders receive cash HA for others/unintended use of the cash	Due to the high poverty and unemployment rates, men may take advantage of powerless categories, people using the cash only for their needs. Buying redundant commodities like Qat, etc.
Safe keep risks/needs more funds towards monitoring and auditing	Cash is a very desirable commodity, and thus, it needs to be monitored to ensure it does not fall into the hands of warring parties or corrupt officials
Prices increase in domestic markets	If prior research was not properly executed, huge cash injections may cause inflation in the economy.

### The Yemeni humanitarian aid projects and INGOs

In response to the ongoing Yemeni humanitarian catastrophe, the World Bank formed the Emergency Crisis Response Project (ECRP) in 2016. The first phase collected US $50 million, to offer temporary jobs and basic commodities. The second phase garnered US $250 million, to tackle starvation among children (World Bank 2018). In 2017, the third phase of the World Bank program raised US $200 million, targeting an additional 1.5 million families (World Bank 2018). Due to its success, the World Bank approved the fourth phase of funds, US $140 million, to keep the program functional in 2018 (World Bank 2018). In total, the World Bank fund was US $640 million, divided into two main cash programs. Firstly, the cash for the emergency program amounted to US $340 million and has been employed by the United Nations Children’s Fund (UNICEF), and secondly, the cash for work program totaled US $300 million and has been employed under the (UNDP) United Nations Development Program (World Bank 2018).

Using the above-assigned funds, INGOs executed multiple cash projects in Yemen, with a variety of goals, targeting different groups of people. Some were designed to provide immediate and unconditional help to people who cannot work and are listed in the Yemeni national social protection program (1.5 million cases). Others were conditional programs, formed to create job opportunities for low-skill workers by contracting with them to build, maintain, and renovate vital and historical infrastructure assets. Moreover, encouraging small handmade business to create stable income projects (Kimball and Jumaan 2020). The following part will display some of those projects and explain their impact on the Yemeni community through the categories of unrestricted and restricted cash projects.

The Emergency Cash Transfer Project ECT project, an unrestricted cash project and UNICEF’s biggest cash-based project, started its implementation in 2017, targeting at meeting people’s basic needs. It was designed to help the neediest individuals, those in vulnerable positions (women/children), and those who cannot work (sick and elders) (UNICEF 2019). The cash distribution is unconditional, empowering people with the choice to buy what they want with “dignity” (UNICEF 2019). The program targeted the pre-conflict recipients of the Yemeni Social Welfare Fund, then it expanded to help those classified with “high-risk and volatile livelihood”. To collect the funds, beneficiaries can go to the closest payment sites out of the thousands of designated places spread all over Yemen, including banks and local shops, and on-ground teams assist those who cannot access those sites (UNICEF 2019). Moreover, the project introduced a messaging platform to communicate with beneficiaries. To ensure the project’s transparency, an independent organization is responsible for the monitoring and compliance during the project’s life cycle (UNICEF 2019). According to the UNICEF, the project helped 1.4 million beneficiaries “indirectly impacting nearly 9 million people—about one third of the country’s population” (UNICEF 2019).

The other type of cash projects adopted in Yemen is a conditional one; cash for work projects differ from digital cash distribution projects, as they link the money distributed with performing a certain task. This allows communities to improve and develop, and it enables individuals to build up their skill sets and have a source of income to meet their basic needs. The project was designed to offer temporary jobs to people with low skills and no sustainable salary, especially in distant areas. It also sought to employ the local human resources to build or maintain the surrounding assets. The project helped multiple sectors; farmers were employed to protect the soil from erosion, improve, and restore agricultural lands to keep the Yemeni agricultural productivity. Others also helped in building wells, roads, and other assets. The cash for work projects have been implemented in Yemen by multiple NGOs targeting different sectors.

### The emergency solid waste management project: during the cholera crisis in Yemen and especially in Hodeidah

The UNDP funded a program, implemented by the Sustainable Development Foundation (SDF), to use paid low-skill labor to help collect solid municipal waste spread around the city (UNDP 2019). Throughout the project’s life cycle in 2019, the total participants were around 2800, receiving YR 3270 (roughly US $6.50) per day for 15–48 working days (UNDP 2019). The project targeted 25,828 households who directly and indirectly benefited from the cash and cleaner environment. “The UNDP so far created 7.1 million employment workdays, reaching 290,000 participants in cash-for-work programs (indirectly benefiting over two million)” (UNDP 2019).

### Promoting livelihood opportunities for urban youth in Yemen

The UNESCO and the European Union have adopted the cash-for-work approach, to firstly, push the Yemeni youth to become actors in the protection and restoration of the heritage sites, especially in Sana’a, Shibam, Zabid, and Aden. The second aim of the project is to “retain traditional skills” and increase cultural public involvement (UNESCO 2019). Other organizations, such as the USAID, adopted similar approaches, the USAID helped 10,700 people through a 1-year rehabilitation cash-for-work infrastructure project in Amran and Hajjah governorates (USAID 2018).

The Yemeni Cash For Work (CFW) project according to the UNDP was spent as follows:

- Nine out of ten used the money to buy food commodities
- One in four used the cash for treatment
- One in five paid back debts

The shift towards cash projects (food assistance) as a substitute or a collaborator to food aid is not custom-made towards Yemen. The World Food Program (WFP), the world’s foremost humanitarian agency, has realized the shortcoming of abundantly depending on food-aid projects. According to the (WFP) “food aid is a tried and tested model, proudly woven into WFP history, it sprang from a largely unidirectional, top-down vision: people were hungry; we fed them”. Food assistance, by contrast, “involves a more complex understanding of people’s long-term nutritional needs and of the diverse approaches required to meet them” (Diouf n.d.). This transformation towards cash interventions in Yemen goes beyond food-aid deficiencies, as they try to tread over generic operational and political issues facing aid providers in Yemen.

### Challenges facing food aid distributors in Yemen

Regularly, INGO’s contract with local NGOs to help in delivering their food aid, giving local NGOs the reign to choose the beneficiaries and the methods of distribution, which in conflict areas divert the aid to align with the “local NGO’s” dogmatic allegiances. In the article “Civil Society During War: the case of Yemen; Elayah,” discusses how Yemeni local NGOs do not operate as an unbiassed side but quite the opposite; they are the palms of factions in the war (2019). In 2014, the estimated number of Yemeni NGOs was 9996, almost doubling in numbers by 2018 to 18,650. Unfortunately, many of those organizations were established by the different partisan actors for their own political interest, varying from, “Hadi forces, the Southern movement and the Houthis” (Elayah and Verkoren 2020) as the number of NGOs in Yemen increased from 9996 in 2014 to 18,650 by 2018, where the different fighting parties use some of those organizations to establish their presence as a “legitimate” party and collect foreign aid through legal channels (Elayah and Verkoren 2020). Consequently, food-aid programs fell in the corruption trap as the World Food Program; announced that 1,200,000 kg of food never reached their destination (Kiley et al. 2019). Also, it was discovered that the food that was supposed to help people in need was being sold by different groups in the secondary market; standardized food packs were being sold with the INGO’s logos on them (Kiley et al. 2019). This was even documented with a video that went viral showing the food supplies being misused in Sarwah, an area controlled by the Houthis in Northern Yemen (MEM 2020). Consequently, the UN suspended a part of its food-aid program to Yemen, as donors pushed to cut UN programs food-aid funds towards Yemen (BBC 2019; Barrington 2020). “The U.N. humanitarian coordinator in Yemen, Lise Grande, told Reuters that 31 of the United Nation’s 41 major programs in Yemen will be reduced or cut in April 2020 without more funding” (Barrington 2020). This was as a result of donors being afraid that maintaining food-aid support with no guarantees to reach its targeted beneficiaries will lengthen the war duration and, which was the case in the South Sudan war when commodities that aimed to reach people suffering were hijacked by different parties, further igniting the war (Kiley et al. 2019). On the other hand, discontinuing the funds towards Yemen because of the misuse of food aid could be catastrophic, especially with the COVID-19 health dilemma. Hence, it became essential for the NGOs to find another option to distribute aid to people in dire need. To increase the number HA operations and limit the amount of aid diverted to the warring parties.

Apart from the administrative problems facing food-aid operators in Yemen, there are some social and economic fundamental shortcomings attached with food aid. Distributing food commodities humiliate people as it neglects their own priorities such as housing and health, it does not change the beneficiary’s situation as “they are only used as a set of passe-partout standard responses in emergencies” (Levine and Chastre 2004). Food aid being under local prices, whether it is free or subsidized, also negatively affects the local food and agricultural market. This consequentially increases the amount of people in need of humanitarian assistance, as farmers and others working the same production cycle lose their sources of income due to the huge flood of imported food commodities, decreasing the local grown products prices and demand (Singer 1988). Some activists have highlighted those developed countries use food-aid programs to spread its “market dominance” with the excess products providing a profit for donor countries (ActionAid 2003). It has found that some countries still keeping it local grain prices steady, removing the extra supply from the market by dumping it as aid. This striving towards market dominance can be observed when the US food aid under the 1954 Public Law 480, which was historically initiated as the “surplus disposal program,” was changed to the more likeable and politically correct name “Food for Peace” (Arestis and Sawyer 2001). Another common criticism of food aid is that it may encourage recipient developing countries to stop working on evolving their national food sector, as they may attain “food dependency” on foreign funds, and furthermore that many emerging nations historically have shown “urban bias” towards deserting crop and cattle production (Arestis and Sawyer 2001, 610).

## Methodology and sample

This research aims to explore the food-aid sustainability and practicality in Yemen against the cash-based interventions, to identify the challenges and deficiencies facing the aid distribution mechanism in Yemen. This comparative study employs the qualitative approach using a purposive sample and secondary data. The primary data was collected through semi-structured interviews, held with Yemeni staff members working at INGOs and NGOs that provide relief through direct implementation of distributing foreign and local aid (*N*=10). The interviews were conducted amid March 2020 and August 2020 among organizations operating or offering aid in Sana’a, Aden, Lahij, Dhale, Shabwah, Hadhramaut, Mahra, Ibb, and Hudaydah governorates in Yemen (*n*=9). These areas are under the control of the three main political powerhouses in Yemen, Hadi forces, the Houthi movement, and the Southern Transitional Council (STC). To evaluate whether the cash transfer approach is suitable for Yemen, the study also interviews beneficiaries of the sampled organizations (*n*=10). Due to the current COVID-19 health situation, in-person interviews were not the only method used; phone or Skype interviews were employed heavily. Full information about this study purpose, method, and use were communicated to the participants before the interviews. Confidentiality concerns were thoroughly conversed as participants asked to keep their personal information from being disclosed. The sample includes a diverse number of participants from the chosen 10 NGOs involved in the entire aid process, from submitting the proposal until delivering the aid to its benefiters. The sample included program officers managing the entire distribution cycle (*n*=3), storekeepers responsible for the warehouses and the physical distribution (*n*=4), staff members working in the administration department from the registering to the verification process (*n*=4), a field researcher surveying the recipients, an auditor monitoring the process, and two consultants advising NGOs on the procedures.

Table 2 illustrates more information about the sample chosen for the purpose of this research.

**Table 2:** Sample and responses

	NGO focus	Location	On ground controlling party	Role	Interview Situation	Interview date (2020)	Gender
1	Relief work	Sana’a City	Houthi	Officer	Phone	8th of June	M
2	Humanitarian assistance	Aden	STC	Storekeeper	Skype	5th of April	F
3	Humanitarian assistance	Lahij	Hadi Forces	Storekeeper	Skype	7th of April	F
4	Humanitarian assistance	Aden	STC	Beneficiary	In person	2nd of July	M
5	Humanitarian assistance	Dhale	Hadi Forces	Storekeeper	Phone	2nd of July	F
6	Humanitarian aid	Sana’a	Houthi	Beneficiary	Phone	12th of August	M
7	Social work	Sana’a	Houthi	Administration	Phone	16th of May	M
8	Relief Work	Sana’a	Houthi	Beneficiary	In person	12th of August	F
9	Economic development	Sana’a	Houthi	Beneficiary	In person	12th of August	F
10	Humanitarian work	Shabwah	Hadi Forces	Consultant	Phone	21 of June	M
11	Humanitarian work	Shabwah	Hadi Forces	Beneficiary	Phone	2nd of July	F
12	Social work	Hadhramaut	Hadi	Officer	Skype	28th of July	M
13	Social Work	Mahra	Hadi	Administration	Phone	25th of May	M
14	Charity	Hudaydah	Hadi Forces	Storekeeper	Phone	27th of July	M
15	Charity	Hudaydah	Hadi Forces	Beneficiary	Phone	27th of July	M
16	Humanitarian aid	Aden	STC	Beneficiary	In person	2nd of July	F
17	Humanitarian aid	Sana’a	Houthi	Beneficiary	Phone	14th of June	M
18	Social work	Sana’a	Houthi	Auditor	Phone	22nd of July	M
19	Humanitarian development	Aden	STC	Field researcher	Skype	8th of August	M
20	Humanitarian aid	Ibb	Houthi	Consultant	Phone	26th of July	M
21	Humanitarian assistance	Sana’a	Houthi	Beneficiary	Phone	17th of June	M
22	Humanitarian assistance	Sana’a	Houthi	Administration	Phone	13th of May	F
23	Humanitarian assistance	Aden	STC	Administration	Phone	28th of July	F
24	Humanitarian assistance	Aden	STC	Officer	Skype	8th of August	M
25	Humanitarian aid	Aden	STC	Beneficiary	In person	2nd of July	F

### The findings: cash as an aid alternative in Yemen

The interviews with the selected sample highlighted multiple issues with the food-aid distribution process, from both the workers and the beneficiaries’ perspectives. The initial findings from the Yemeni food-aid mechanism case study are divided into two sections; the organizational distribution issues by the various different NGOs and the complaints raised by the recipients to the sample organizations regarding the lack of diversification and low quality of food products. The primary research showed that the diversion and misuse of food aid typically happens during the verification and distribution process. Usually, the initial recipients list prepared by the organizations are accurate and include the names of people in dire need of help, as members of the donor organizations regularly supervise the procedure. However, because of the defacto-governmental pressure and the logistical obstacles such as the change/delay in delivery timings, transportation costs, or individual’s health conditions, recipients are hindered from attending the distribution process and food aid gets averted. According to the interviewees, the diversion of aid occurs due to the political pressure or security threats targeted at the NGO staff members. Aid workers are pushed by warring parties to divert the food baskets to certain members affiliated with the defacto-parties, who use it for economic or political gain. The interviews have shown that mostly organization working under the Houthi-controlled areas complained about the political pressure influencing their aid operations. From a logistical perspective, when a recipient fails to attend during the distribution process, the names on the beneficiaries’ list get modified with different recipients by NGO members responsible for registering and verification process. However, many interviewees stated that people are being added without checking if they are in need for the food supplies, and sometimes they add people from within their close circle, such as family members or friends, who will resell it for personal gain. Food supplies used to be distributed on certain dates, and once a recipient has not shown up, they remove his name from the list and put someone they know instead, even if they do not meet the criteria of individuals who deserve the food baskets supply. Another issue is that once the distribution cycle ends and extra food bags are available, they try to discard them by any means “sometimes even to random people on the street”. This happens to make sure that they get another full patch from the donor organization and to avoid the expiration of certain products. One more observation another staff member added is that there will be a truck next to the distribution warehouse buying the supplies from the recipients once they collect their share; he added that they do this in front of the staff members and that the only commodity beneficiaries keep is the cooking oil. Moreover, they add that local shop owners have complained that they cannot sell their products anymore as food-aid goods have been flooding the market, pushing them to use their shops to resell food-aid products because, although they are at lower quality, they are below the average market prices. This has led to the abolishment of local food manufacturers and market share, contributing to the already catastrophic economy dilemma. From the interviews, we noticed that when the donors are local, the diversion of food tend to be less than the usual as they closely supervise the distribution process and have fewer predetermined beneficiaries compared to the international donors.

The aid recipients mostly complained about the quality of the products as the grains were usually in below average conditions, and the products were always repetitive. This has led them to frequently store extra quantities of products they have an abundance of and are not going to use; so, they end up selling or exchanging them for other products. Furthermore, recipients continuously complained about the transport costs as they cannot use local transportation to take home their products; car rental prices have been high and were for some beneficiaries around 50% of the price of resold aid supplies. This humiliates people as sometimes they cannot sell the food items and even if they do their underpriced. The beneficiaries stated that they head towards reselling their assigned food-aid products as soon as possible, to meet with other crucial needs such as paying for medical bills and rent.

On the other hand, cash aids’ biggest advantage is that it empowers people to decide on their priorities and increase their purchasing power, to have a healthier diet, housing, medical bills, education, pay back debts, and in some cases save up to start small business (Overseas Development Institute (ODI) and the Center for Global Development (CGD) 2015). The restricted and unrestricted cash projects have helped to improve people’s dignity as they have enabled higher access to more life essentials. However, the primary research showed that in some underprivileged cases, the little amounts of money offered were not enough to provide the basic living conditions required and by default has not enhanced their dignity. Thus, currently cash aid has not worked as intended; hence, more research is needed to reach the correct amounts that will significantly improve people’s livelihoods, in a respectable manner.

Nevertheless, the major concern that all participants share was the fear of donor organizations stopping or minimizing the amount of aid towards Yemen. Donors have lost their trust in the HA distribution process and halted funds due to the project’s deficiencies and misappropriation. With this, NGOs working in Yemen desperately need to avoid new scandals by offering a secure and effective method to support the Yemeni population, while maintaining the flow of humanitarian funds. Cash-based projects as an aid alternative, accompanied with a digital trackable method, offers a swift way to overcome the diversion of aid by the war parties. It has become essential to gain back the trust of UN donor members to remobilize the funds towards Yemen. The distribution of cash aid through rigorous and various digital payments offers a fast, flexible, secure, and discrete aid distributing channels with beneficiaries (Overseas Development Institute (ODI) and the Center for Global Development (CGD) 2015). It allows for better control by reducing the ability of NGOs to modify the primary recipients lists, reducing and eliminating the risk of third-party assistance confiscation, by minimizing the physical circulation of aid. It also offers beneficiaries a simpler and cheaper way to receive the aid by removing the transportation costs they had to bear every distribution cycle. Moreover, conditional cash projects funds, being tied with individuals preforming certain tasks, enables local residents to build essential infrastructural projects that can be used by the surrounding community. Cash-for-work projects also tackle the assumption that people capable of work will get lazy and dependent once they start getting cash as aid, leading to high levels of unemployment. NGOs would still need to monitor spending patterns, inform people about digital payments, and reach those who do not have the technology or access.

Allocating funds towards cash aid contrary to food aid also has an economic advantage due to the lower logistical costs. Saving up on the operational costs by physical cash exchange or digital transfers should by default increase the impact of the cash-based projects to reach more people than the food-aid projects. This was the case in Somalia, where only 35% of the actual food-aid funds reached the beneficiaries, compared to 85% that reached the cash-aid recipients (Hedlund et al. 2013; Overseas Development Institute (ODI) and the Center for Global Development (CGD) 2015). Moreover, cash-based aid has a higher percentage of impact on the society, as shown by the UNICEF project, which was directed at 1.4 million recipients and another nine million people lives were positively affected, while the scale of impact of food-aid projects is limited to the targeted population. Increasing the amount of cash supply in the market will help decrease the economy stagnation, through growing people’s purchasing power. This will by default increase the market demand and supply of different products, pushing the private sector to be involved in providing enough supplies of necessities and increasing the local production cycle, creating jobs, and investments and indirectly benefiting a larger scale of people. However, it is important to note that it could also cause inflation because as incomes rise so does the cost of living, unless measures are put in place to control this increase.

Although all the participants agreed on the superiority and effectiveness of cash aid over food-aid projects, cash-aid projects are still small scale compare to the food aid larger scale projects in Yemen. The staff members claimed that local NGOs prefer to work with food aid, as they have a higher funding approval rate, being a tested model that they have experience with and that is easier to justify during the proposals phase than cash projects. A staff member added that the NGO’s “supply and demand mentality” has pushed many of them to shift their purpose and activities towards food-aid distribution programs. This is especially the case given that almost all aid funds offered by the donors nowadays are fixated towards emergency humanitarian projects in a form of food aid. However, due to the adversities NGOs face with the misappropriations of food-aid supplies, Yemen is now fronting the danger of donors cutting the funds greatly needed by the Yemeni population, especially with the current COVID-19 health situation. Hence, cash projects should no longer be the anomaly in Yemen, the international community need to start considering cash—aid instead of food aid, as it has been proved that it can be an efficacious method in conflict countries. Adopting more cash projects in Yemen would also decrease local NGO’s role in direct aid distribution, encouraging them to focus on sustainable development projects, education, women rights, and other pressuring matters.

## Conclusion and recommendations

The Yemeni population is facing the worst humanitarian crisis in the world. The massive food-aid programs in Yemen have marginally helped decrease the severity of the hunger situation, but they have created a bigger dilemma with donor’s threatening to stop the flow of funds, due to the diversion of food aid by the defacto parties resulting in food aid becoming a weapon of war (Barrington 2020). Thus, it has become essential to gain back the donor’s trust and maintain the flow of funds, offering cash aid as a superior method to food aid in Yemen, capable of decreasing the deviation of aid. Adopting more cash-aid programs does not imply eliminating food-aid projects. Both cash and food aid cannot ommit all the risks invloved during the distibution of aid. But cash aid offers a way to mitagte the aid distribution risks in Yemen, especially that the war ongoing since 2014, calling for long-term options instead of the widly used short-term HA methods. Cash-based intervention effects are long term when compared with food aid, as they aim to “alleviate hunger here and now with the broader objective of ending hunger once and for all” (Diouf n.d.), aligning with the UN’s first and second sustainable development goals (SDGs) to eliminate poverty and hunger. Cash aid has proved to be more efficient than food interventions in conflict countries such as “Somalia where 2.5 more of aid budgets went directly to beneficiaries with cash than with food aid” (Overseas Development Institute (ODI) and the Center for Global Development (CGD) 2015). However, for cash-aid projects to work in Yemen as Somalia, cash-aid projects need to be integrated within a trackable electronic cash transfer system that can be implemented through customized NGO systems such as “The Electronic Cash Transfer Learning Action Network, convened by Mercy Corps” (Overseas Development Institute (ODI) and the Center for Global Development (CGD) 2015) or by collaborating with the private sector. Cash aid will aim to either mobilize the existing various currency transfer platforms in Yemen offered by money transfer companies and banks or create a new platform with the support of the mobile network companies. Adopting the digital payment methods during humanitarian crises in a war-torn country has already proved its success in the case of Somalia, where it reached more than a million recipients (Overseas Development Institute (ODI) and the Center for Global Development (CGD) 2015). In addition, the digital distribution of cash aid if implemented correctly could be the solution for the global lack of data concerning the real amount of aid reaching its destined recipients. Although this information would help donors to more efficiently “monitor, evaluate, and communicate the aid projects results with both the affected populations and the tax-paying public in donor countries” (Overseas Development Institute (ODI) and the Center for Global Development (CGD) 2015). The data protection risks increase so the NGOs need to make sure to employ a secure “closed loop system” that must be “collected, managed, stored, and shared in line with good data management practices” (Burton 2020, p 56). Morover, using digtal platforms is not the only feasible way to distribute cash aid as creating functional, secure elactronic system for the HA in Yemen will take time. Multiple methods to distibute cash can be eployed at the same time where this was done by the the ICRS in the Democratic Republic of the Congo. The ICRS uses three methods: “cash payments directly to people, digital payments via mobile money, and digital payments via personal accounts held with cooperatives”” (Burton 2020, p 53).

Nevertheless, for the cash-aid projects to be successful in helping the eight million whom lost their source of income and the two million internally displaced Yemenis, once implemented, unrestricted cash-aid packages should not be standardized. They should take into consideration the number of family members, the health of individuals, housing, and other economic and social factors. Conditional cash programs should include skill training for its participants and offer them a way to earn a decent income after the projects end, such as carpentry and pluming and grants to allow the local people to set up small businesses. The list of recipients should be audited regularly by either the donor organization or a third party or secure digitalized verifications should be used, such as a fingerprint or face ID methods that can only be accessed by the designated person. Lastly, the NGOs need to track the spending patterns to prevent socially destructive habits and protect marginalized beneficiary groups from being exploited, by offering them a variety of aid delivery methods. It will also help to track the spending patterns to assess whether different projects have reached their objectives.

After discussing the challenges facing NGOs in conflict areas, examining empirical information about cash transfers as an alternative aid mechanism and drawing on the experience from different countries with complex circumstances against the food-aid program in Yemen, this research concludes that cash-based interventions during emergencies offer a great alternative as an aid mechanism. This method targets a wider scope of peoples’ necessities, in a more efficient and effective delivery approach that can be easily tracked by the donors. Hence, a significant proportion of the huge funds allocated for food aid in Yemen should be relocated towards cash aid when NGOs are able to provide the required conditions to ensure significant success and impact. Further studies are required in this field and should be applied to different conflict states, as more primary information would enrich this topic, adding more information to close the gaps.

## Data Availability

Not applicable.

## Footnotes

1 Doocy, S. (2017). The effectiveness and value for money of cash-based humanitarian assistance: a systematic review. *Journal of Development Effectiveness*, 10(1), pp.121-144.

2 Forsythe, D. P. (2005). The humanitarians: The international committee of the Red Cross. Cambridge University Press, 16.

3 Development Initiatives’ Report (2019), available at http://devinit.org/wp-content/uploads/2019/09/GHA-report-2019.pdf
